# Low 1,5-anhydroglucitol levels are associated with long-term cardiac mortality in acute coronary syndrome patients with hemoglobin A1c levels less than 7.0%

**DOI:** 10.1186/s12933-017-0636-1

**Published:** 2017-11-21

**Authors:** Shohei Ouchi, Kazunori Shimada, Tetsuro Miyazaki, Shuhei Takahashi, Yurina Sugita, Megumi Shimizu, Azusa Murata, Tomoyasu Kadoguchi, Takao Kato, Tatsuro Aikawa, Shoko Suda, Eiryu Sai, Masaru Hiki, Hiroshi Iwata, Takatoshi Kasai, Katsumi Miyauchi, Hiroyuki Daida

**Affiliations:** Department of Cardiovascular Medicine, Juntendo University Graduate School of Medicine, 2-1-1 Hongo Bunkyo-ku, Tokyo, 113-8421 Japan

**Keywords:** 1,5-anhydroglucitol, Diabetes mellitus, Postprandial hyperglycemia, Acute coronary syndrome, Cardiac mortality

## Abstract

**Background:**

Diabetes mellitus is considered an important risk factor for cardiovascular diseases. High hemoglobin A1c (HbA1c) levels, which indicate poor glycemic control, have been associated with occurrence of cardiovascular diseases. There are few parameters which can predict cardiovascular risk in patients with well-controlled diabetes. Low 1,5-anhydroglucitol (1,5-AG) levels are considered a clinical marker of postprandial hyperglycemia. We hypothesized that low 1,5-AG levels could predict long-term mortality in acute coronary syndrome (ACS) patients with relatively low HbA1c levels.

**Methods:**

The present study followed a retrospective observational study design. We enrolled 388 consecutive patients with ACS admitted to the cardiac intensive care unit at the Juntendo University Hospital from January 2011 to December 2013. Levels of 1,5-AG were measured immediately before emergency coronary angiography. Patients with early stent thrombosis, no significant coronary artery stenosis, malignancy, liver cirrhosis, a history of gastrectomy, current steroid treatment, moderately to severely reduced kidney function (estimated glomerular filtration rate < 45 ml/min/1.73 m^2^; chronic kidney disease stage 3B, 4, and 5), HbA1c levels ≥ 7.0%, and those who received sodium glucose co-transporter 2 inhibitor therapy were excluded.

**Results:**

During the 46.9-month mean follow-up period, nine patients (4.5%) died of cardiovascular disease. The 1,5-AG level was significantly lower in the cardiac death group compared with that in the survivor group (12.3 ± 5.3 vs. 19.2 ± 7.7 µg/ml, *p* < 0.01). Kaplan–Meier survival analysis showed that low 1,5-AG levels were associated with cardiac mortality (*p* = 0.02). Multivariable Cox regression analysis showed that 1,5-AG levels were an independent predictor of cardiac mortality (hazard ratio 0.76; 95% confidence interval 0.41–0.98; *p* = 0.03).

**Conclusion:**

Low 1,5-AG levels, which indicate postprandial hyperglycemia, predict long-term cardiac mortality even in ACS patients with HbA1c levels < 7.0%.

## Background

Diabetes mellitus (DM) is considered an important risk factor for cardiovascular disease, including acute coronary syndrome (ACS), and death [1–5]. High hemoglobin A1c (HbA1c) levels, which indicate poor glycemic control, have been associated with poor prognosis in patients with diabetes mellitus [6–10]. Treatment of DM is performed using HbA1c as an indicator, and an HbA1c level < 7.0% is the recommended target for adult patients as per the American Diabetes Association and the European Association for the Study of Diabetes [11, 12]. However, previous studies have shown that atherosclerosis is pre-existent even at a stage when impaired glucose tolerance and postprandial hyperglycemia are detected [13–15]. Indeed, numerous studies have reported cardiovascular diseases, including ACS, in patients with HbA1c < 7.0% [13–16].

1,5-Anhydroglucitol (1,5-AG) is a monosaccharide found in most foods [17]. The concentration of 1,5-AG in blood and tissues is maintained constant due to reabsorption in the renal proximal tubule [17, 18]. As excretion of glucose into the urine increases, reabsorption of 1,5-AG is inhibited competitively and the blood level of 1,5-AG decreases. Therefore, low 1,5-AG levels in blood are considered a clinical marker of postprandial hyperglycemia [19, 20].

There are few parameters which can predict cardiovascular risk in patients with well-controlled diabetes and/or with relatively low HbA1c levels. We and other groups have reported that 1,5-AG levels are associated with atherosclerotic disease, including coronary artery disease and risk of revascularization after elective percutaneous coronary intervention [20–23]. Therefore, we hypothesized that low 1,5-AG levels could predict long-term mortality in ACS patients with relatively low HbA1c levels.

## Methods

### Patients

**Fig. 1:**
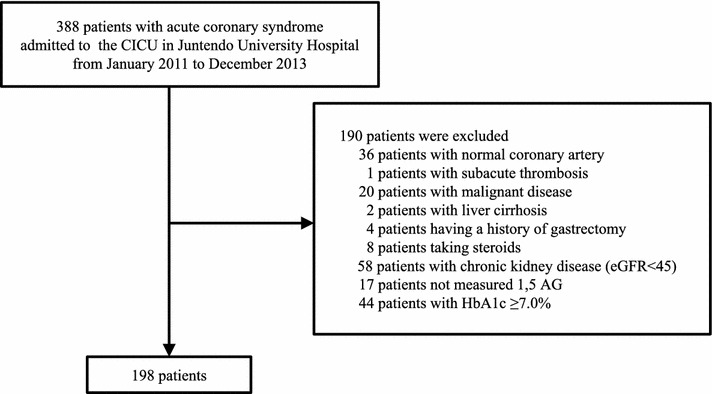
Flow diagram of the study subjects

The present study was conducted as a retrospective observational study. All subjects gave their informed consent for inclusion before they participated in the study. The study was conducted in accordance with the Declaration of Helsinki and the protocol was approved by the Ethics Committee of Juntendo University Hospital. First, we recruited 388 consecutive patients with ACS admitted to the cardiac intensive care unit at the Juntendo University Hospital between January 2011 and December 2013. Patients with early stent thrombosis (occurring 0–30 days post stent implantation) [24], no significant coronary artery stenosis, malignancy, liver cirrhosis, a history of gastrectomy, current steroid treatment, moderately to severely impaired kidney function (defined as an estimated glomerular filtration rate (eGFR) of < 45 ml/min/1.73 m^2^; chronic kidney disease stage 3B, 4, and 5 [25]), HbA1c levels ≥ 7.0%, and those under treatment with sodium-glucose co-transporter 2 inhibitors were excluded from the study. Study subject selection is depicted in Fig. 1.

### Definitions

ACS was defined as presence of unstable angina pectoris, non-ST elevation myocardial infarction, or ST elevation myocardial infarction. Unstable angina pectoris was defined as angina at rest or in an accelerating pattern with negative cardiac biomarkers with or without ECG changes indicative of myocardial ischemia (ST segment depression or transient elevation or new T wave inversion) [26]. Non-ST elevation myocardial infarction was defined as an increase (≥ 2-fold) in serum creatine kinase, and troponin T positivity, without ECG changes of persistent ST-elevation [26]. ST elevation myocardial infarction was defined as an increase (≥ 2-fold) in serum creatine kinase, and troponin T positivity, with ECG changes of persistent ST-elevation [27]. There were 59 patients with unstable angina pectoris, 53 patients with non-ST elevation myocardial infarction, and 86 patients with ST elevation myocardial infarction. Cardiac death was defined as death due to cardiovascular disease (e.g., heart failure, myocardial infarction, sudden death). Multi-vessel coronary disease was defined as presence of coronary artery disease of two or more vessels with visually assessed stenosis of more than 75% based on the American Heart Association Classification [28, 29]. DM was defined as presence of a previous diagnosis in medical records, HbA1c levels of ≥ 6.5%, or treatment with oral anti-diabetic agents or insulin. Dyslipidemia was defined as presence of a previous diagnosis in medical records, abnormal lipid profiles (i.e., triglyceride levels ≥ 150 mg/dl, low-density lipoprotein cholesterol levels ≥ 140 mg/dl, or high-density lipoprotein cholesterol levels ≤ 40 mg/dl), or treatment with anti-dyslipidemic agents. Hypertension was defined as presence of a previous diagnosis due to systolic blood pressure ≥ 140 mmHg and/or diastolic blood pressure ≥ 90 mmHg in medical records, or treatment with anti-hypertensive agents [30].

### Data collection and blood sampling

Clinical findings including age, gender, body mass index, smoking, family history of coronary artery disease, blood pressure, heart rate, left ventricular ejection fraction, and medical history of DM, dyslipidemia, hypertension, atrial fibrillation, and revascularization were collected on admission. Laboratory findings of total cholesterol, triglycerides, high-density lipoprotein cholesterol, low-density lipoprotein cholesterol, eGFR, brain natriuretic peptide, albumin, hemoglobin, and HbA1c were obtained after an overnight fast, within 24 h of admission. The eGFR was calculated using the Japanese equation comprising the serum creatinine level, age, and gender as follows: eGFR (ml/min/1.73 m^2^) = 194 × creatinine^−1.094^ × age^−0.287^ (for females, × 0.739) [31]. Plasma total cholesterol and creatinine levels were measured using enzymatic methods, triglyceride levels using visible absorption spectrometry, high-density lipoprotein cholesterol levels using the direct method, and low-density lipoprotein cholesterol levels were calculated using the Friedewald formula [32]. Brain natriuretic peptide levels were measured using the one-step sandwich enzyme immunoassay method, albumin levels using the bromocresol purple method, hemoglobin levels using the cyanmethemoglobin method, and HbA1c levels using high-performance liquid chromatography. Blood samples were obtained immediately prior to coronary angiography and stored at − 80 °C until measurement of 1,5-AG levels. Serum 1,5-AG levels were measured with a colorimetric method (Nippon Kayaku, Tokyo, Japan) using a Lana 1,5-AG auto liquid automatic analyzer (JCA-BM 8060, JEOL Ltd., Tokyo, Japan).

### Angiographical analysis

Coronary artery angiography was performed in all patients during hospitalization. We performed angiographical analysis for all patients in order to evaluate coronary artery disease and/or thromboembolic disorders. Significant stenosis was defined as a decrease in the pre-stenotic diameter of the blood vessels > 75% in the left anterior descending coronary artery, left circumflex coronary artery, and right coronary artery, and > 50% in the left main coronary trunk. Quantitative coronary angiography was performed in all subjects, and analyses were performed by a technician without any knowledge of the study design [33]. All treatment decisions including use of bare metal stents, drug eluting stents, or balloon alone for percutaneous coronary intervention, and choice of coronary artery bypass grafting or drug therapy alone were made at the discretion of the physician.

### Statistical analysis

Continuous variables were represented as means with standard deviations, and categorical variables were represented as counts and percentages. Comparisons of continuous variables were performed using the Student’s *t*-test or the Mann–Whitney *U* test. Categorical variables were analyzed using the Chi squares test or the Fisher’s exact probability test. Unadjusted cumulative event rate for the primary endpoint was estimated using the Kaplan–Meier method and was compared between groups using the log-rank test. The cut-off value was defined using the median of the 1,5-AG level (18.5 µg/ml). Univariate and multivariate Cox regression analyses were performed to identify the predictor of the primary endpoint. Hazard ratios (HRs) and 95% confidence intervals (CIs) were also calculated. Age, gender, body mass index, and all variables with a *p*-value < 0.1 in univariate Cox regression analysis were included in the multivariate analyses. JMP12 (for Windows, SAS Institute, Cary, NC) was used for statistical analyses, and *p*-values < 0.05 were considered statistically significant.

## Results

Of the 388 patients enrolled in the study, 198 patients were examined. The mean patient age was 65 ± 12 years and 164 patients (82.8%) were male. The patients were followed up until December 2016. The mean follow-up duration was 46.9 ± 16.9 months, and the maximum follow-up duration was 72.7 months. During follow-up, 9 patients (4.5%) had cardiovascular death (sudden death: 4 patients, heart failure: 5 patients). These death events were retrieved from the hospital medical records. The patients were categorized into the survivor group or the cardiac death group. Patient characteristics and laboratory findings are shown in Table 1. The 1,5-AG level was significantly lower in the cardiac death group than in the survivor group (12.3 ± 5.3 vs. 19.2 ± 7.7 µg/ml, *p* < 0.01). Patients in the cardiac death group were significantly older, had lower levels of left ventricular ejection fraction, albumin, and hemoglobin, and had significantly higher levels of brain natriuretic peptide and HbA1c than those in the survivor group (*p* < 0.05 in all comparisons).

**Table 1:** Patient characteristics and laboratory findings at hospitalization

	Survivor group (n = 189)	cardiac death group (n = 9)	*p*-value*
Age (years)	65 ± 12	75 ± 12	< 0.01
Male, n (%)	156 (82.5)	8 (88.9)	NS
Body mass index (kg/m^2^)	24.3 ± 3.3	22.4 ± 2.4	NS
Diabetes mellitus, n (%)	34 (18.0)	3 (33.3)	NS
Dyslipidemia, n (%)	152 (80.4)	7 (77.8)	NS
Hypertension, n (%)	103 (54.5)	5 (55.6)	NS
Current smoker, n (%)	63 (33.3)	1 (11.1)	NS
Family history of CAD, n (%)	58 (30.7)	1 (11.1)	NS
Atrial fibrillation, n (%)	18 (9.5)	2 (22.2)	NS
History of revascularization			
PCI, n (%)	31 (16.4)	1 (11.1)	NS
CABG, n (%)	7 (3.7)	0 (0.0)	NS
Systolic blood pressure (mmHg)	139 ± 26	139 ± 36	NS
Diastolic blood pressure (mmHg)	79 ± 19	79 ± 24	NS
Heart rate (/min)	77 ± 17	88 ± 24	NS
LVEF (%)	56.6 ± 11.7	47.7 ± 14.0	0.03
Total cholesterol (mg/dl)	187 ± 43	168 ± 43	NS
Triglycerides (mg/dl)	137 ± 95	108 ± 33	NS
HDL-cholesterol (mg/dl)	46 ± 12	41 ± 15	NS
LDL-cholesterol (mg/dl)	115 ± 39	105 ± 28	NS
eGFR (ml/min/1.73 m^2^)	81.3 ± 20.4	68.1 ± 17.2	NS
Brain natriuretic peptide (pg/ml)	151 ± 345	597 ± 882	0.03
Albumin (g/dl)	4.0 ± 0.5	3.6 ± 0.7	0.01
Hemoglobin (g/dl)	14.5 ± 1.7	13.0 ± 2.3	0.01
Hemoglobin A1c (%)	5.8 ± 0.5	6.2 ± 0.6	0.02
1,5-AG (µg/ml)	19.2 ± 7.7	12.3 ± 5.3	< 0.01

Data are presented as mean ± standard deviation, or number (percentage)

*CAD* coronary artery disease, *PCI* percutaneous coronary intervention, *CABG* coronary artery bypass grafting, *LVEF* left ventricular ejection fraction, *HDL* high-density lipoprotein, *LDL* low-density lipoprotein, *eGFR* estimated glomerular filtration rate, 1,5-*AG* 1,5-anhydroglucitol

* Comparisons between the survivor and the cardiac death groups

Affected coronary artery status, treatment strategy, and pharmacological therapy at discharge are shown at Table 2. Diuretic administration at discharge was significantly more common in the cardiac death group than in the survivor group (*p* = 0.03).

**Table 2:** Affected coronary artery status, treatment strategy, and pharmacological therapy at discharge

	Survivor group (n = 189)	Cardiac death group (n = 9)	*p* **-**value*
Number of diseased vessel			NS
One, n (%)	80 (42.3)	2 (22.2)	
Two, n (%)	54 (28.6)	3 (33.3)	
Three, n (%)	55 (29.1)	4 (44.5)	
Stenosis of LMT, n (%)	15 (7.9)	2 (22.2)	NS
Multi-vessel, n (%)	112 (59.3)	8 (88.9)	NS
Target lesion			NS
LAD, n (%)	97 (51.3)	4 (44.5)	
LCX, n (%)	32 (16.9)	0 (0.0)	
RCA, n (%)	53 (28.1)	3 (33.3)	
LMT, n (%)	7 (3.7)	2 (22.2)	
Treatment strategy			NS
PCI (BMS), n (%)	82 (43.4)	4 (44.5)	
PCI (DES), n (%)	64 (33.9)	3 (33.3)	
POBA, n (%)	14 (7.4)	0 (0.0)	
CABG, n (%)	12 (6.3)	1 (11.1)	
Pharmacological therapy at discharge			
Diuretics, n (%)	39 (20.6)	5 (55.6)	0.03
Anti-platelets, n (%)	188 (99.5)	9 (100)	NS
Anti-coagulants, n (%)	15 (7.9)	2 (22.2)	NS
CE inhibitor or ARB, n (%)	134 (70.9)	7 (77.8)	NS
Beta-blocker, n (%)	133 (70.4)	6 (66.7)	NS
Calcium-channel blocker, n (%)	52 (27.5)	1 (11.1)	NS
Statin, n (%)	171 (90.5)	6 (66.7)	NS
Oral hypoglycemic agents, n (%)	20 (10.6)	2 (22.2)	NS
Sulfonylurea, n (%)	5 (2.7)	1 (11.1)	NS
Alpha-glucosidase inhibitor, n (%)	9 (4.8)	0 (0.0)	NS
DPP-4 inhibitor, n (%)	8 (4.2)	2 (22.2)	NS
Metformin, n (%)	4 (2.1)	0 (0.0)	NS
Pioglitazone, n (%)	6 (3.2)	0 (0.0)	NS
Glinide, n (%)	0 (0.0)	0 (0.0)	NS
Insulin, n (%)	2 (1.1)	0 (0.0)	NS
Inotropic agents, n (%)	4 (2.1)	0 (0.0)	NS

Data are presented as number (percentage)

*LMT* left main coronary trunk, *LAD* left anterior descending coronary artery, *LCX* left circumflex coronary artery, *RCA* right coronary artery, *PCI* percutaneous coronary intervention, *BMS* bare metal stent, *DES* drug eluting stent, *POBA* percutaneous old balloon angioplasty, *CABG* coronary artery bypass grafting, *ACE* angiotensin converting enzyme, *ARB* angiotensin-II receptor blocker, *DPP*-*4* dipeptidyl peptidase-4

* Comparisons between the survivor and the cardiac death groups

In this study population, the 1,5-AG level was negatively related to age, brain natriuretic peptide level, and HbA1c level (*p* < 0.05 in all comparisons). Furthermore, 1,5-AG levels were significantly lower in patients with DM, hypertension, multi-vessel disease, left main coronary trunk lesions, or anti-platelet or oral hypoglycemic agent administration at discharge, than in their counterparts (*p* < 0.05 in all comparisons). The 1,5-AG levels were significantly higher in current smokers than non-smokers (*p* = 0.02).

**Fig. 2:**
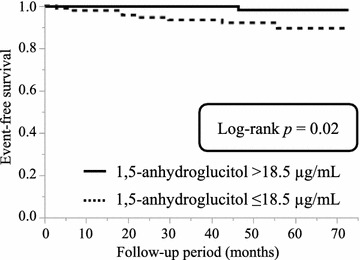
Event-free survival curve for cardiac death in patients with acute coronary syndrome. Unadjusted cumulative event rate for the primary endpoint (cardiac death) was estimated using the Kaplan–Meier method and compared between groups using the log-rank test. We defined the cut-off value as the median level of 1,5-anhydroglucitol (18.5 µg/ml)

Kaplan–Meier analysis was performed to estimate the unadjusted event-free rate of cardiac death. Patients were categorized into two groups based on median 1,5-AG level (18.5 µg/ml). Event-free survival rate was significantly lower in the low 1,5-AG group than in their counterparts with high 1,5-AG (*p* = 0.02, Fig. 2).

Univariate Cox regression analyses revealed that 1,5-AG level, age, gender, left ventricular ejection fraction, HbA1c level, eGFR, brain natriuretic peptide level, albumin level, hemoglobin level, multi-vessel disease, and diuretic or statin administration at discharge were associated with long-term cardiac mortality. After accounting for confounding variables, 1,5-AG was found to be significantly associated with long-term cardiac mortality (1 μg/ml increase; HR, 0.76; 95% CI, 0.41–0.98; *p* = 0.03, Table 3).

**Table 3:** Univariate and multivariate Cox regression analyses of cardiac death

	Univariate			Multivariate		
	HR	95% CI	*p*-value	HR	95% CI	*p*-value
Age, 1 year increase	1.10	1.03–1.18	< 0.01	1.25	1.06–1.65	< 0.01
Male, yes	1.51	0.28–28.0	0.7	0.07	0.01–4.76	NS
Body mass index, 1 kg/m^2^ increase	0.83	0.66–1.03	0.09	0.89	0.57–1.28	NS
LVEF > 60%, yes	0.13	0.01–0.72	0.02	0.02	0.01–0.42	0.01
HbA1c, 0.1% increase	1.17	1.03–1.34	0.02	1.13	0.92–1.42	NS
eGFR, 1 ml/min/1.73 m^2^ increase	0.96	0.92–0.99	0.04	0.88	0.76–0.97	< 0.01
BNP > 100 pg/ml, yes	4.01	1.06–19.0	0.04	0.32	0.01–5.20	NS
Albumin, 0.1 g/dl increase	0.86	0.77–0.98	0.02	1.13	0.90–1.54	NS
Hemoglobin, 1 g/dl increase	0.65	0.46–0.92	0.02	1.06	0.57–2.16	NS
Multi-vessel disease, yes	5.28	0.97–98.0	0.06	5.52	0.07–2710	NS
Diuretic usage, yes	4.60	1.22–18.6	0.03	0.17	0.01–2.19	NS
Statin usage, yes	0.23	0.06–1.09	0.06	0.004	0.01–0.11	< 0.01
1,5-AG, 1 µg/ml increase	0.88	0.79–0.97	< 0.01	0.76	0.41–0.98	0.03

*LVEF* left ventricular ejection fraction, *HbA1c* hemoglobin A1c, *eGFR* estimated glomerular filtration rate, *BNP* brain natriuretic peptide, 1,5-*AG* 1,5-anhydroglucitol

## Discussion

The present study demonstrated that low 1,5-AG levels, but not HbA1c levels, could predict long-term mortality in ACS patients with HbA1c level < 7.0%.

Treatment of DM in adult patients is done with a goal to achieve HbA1c level < 7.0%, which is associated with prevention of DM-associated complications. However, macrovascular disease cannot be completely prevented even under the above treatment conditions [34–39]. The United Kingdom prospective Diabetes Study (UKPDS) showed that intensive blood-glucose control decreases risk of microvascular complications by 25%, but not that of macrovascular disease in patients with type 2 DM. Further, all intensive treatments were found to increase the risk of hypoglycemia [34]. However, during the 10-year post-trial follow up, a continuous decrease in microvascular complications, risk of all-cause death, and myocardial infarction were seen over time in the intensive blood-glucose control group [40]. The Action to Control Cardiovascular Risk in Diabetes (ACCORD) study demonstrated that the use of intensive therapy to target normal HbA1c levels increased mortality and did not significantly reduce major cardiovascular events as compared with standard therapy [35]. The Action in Diabetes and Vascular Disease: Preterax and Diamicron Modified Release Controlled Evaluation (ADVANCE) showed that a strategy of intensive glucose control lowered HbA1c to 6.5% and reduced the incidence of microvascular events, but did not significantly reduce the incidence of major macrovascular events or death [36]. Comprehensive evaluation in addition to that of HbA1c may be necessary for prevention of macrovascular disease, especially in patients with HbA1c levels < 7.0%.

The HbA1c level reflects the average blood glucose level over the past 2 months and can be confounded in patients with anemia, hemoglobinopathy, and renal dysfunction [41]. The 1,5-AG level is more reflective of short-term glycemic status compared with the HbA1c level. Moreover, 1,5-AG level reflects postprandial hyperglycemia and glycemic variability, which are not captured by HbA1c measurement [19, 42].

A previous cohort study reported that postprandial hyperglycemia was associated with cardiovascular disease and death [43, 44]. The Diabetes Epidemiology: Collaborative analysis of Diagnostic criteria in Europe (DECODE) study showed that postprandial hyperglycemia was a better predictor of mortality in subjects with newly diagnosed DM and in those with impaired glucose tolerance than fasting glucose [43]. Previous studies have also reported that 1,5-AG levels were associated with atherosclerosis and cardiovascular disease [22, 45–48]. Fujiwara et al. reported that 1,5-AG was associated with the presence of de novo coronary artery disease in 227 consecutive patients with HbA1c levels < 7.0% [46]. Selvin et al. reported that among 11,106 participants in the Atherosclerosis Risk in Communities study, subjects with DM and 1,5-AG levels < 6.0 µg/ml had an increased risk of coronary artery disease, stroke, heart failure, and death compared to subjects with 1,5-AG levels ≥ 6.0 µg/ml and no history of DM [47]. In the Suita study, a cohort study in Japan, Watanabe et al. reported that adjusted HRs of all cardiovascular diseases in men increased linearly with decreasing 1,5-AG, and that HR was 2.22 in the category with lowest 1,5-AG (≤ 14.0 µg/ml) compared to the category with highest 1,5-AG (≥ 24.5 µg/ml) in 2095 initially healthy Japanese men without a history of coronary artery disease or stroke [48]. We have recently reported that low and exacerbated levels of 1,5-AG were associated with cardiovascular events in patients with HbA1c < 7.0% who underwent first-time elective percutaneous coronary intervention [23]. The present study is the first to report that 1,5-AG levels are significantly associated with cardiac mortality in ACS patients with HbA1c < 7.0%.

The clear cut-off value of 1,5-AG for diagnosis of DM remains unclear. This study population and events were relatively small, therefore, the cut-off value was defined using the median of 1,5-AG levels. The 1,5-AG levels are reported to be influenced by diet and race and are relatively lower in the Japanese than in Americans [41, 49]. Further studies are required in order to determine the cut-off values for cardiovascular events in patients with different conditions and of various races [50].

This study had several limitations. First, the study was conducted in a single institution and the study population was relatively small. Studies with a larger sample size and racial diversity will be more effective in evaluating the association between 1,5-AG levels and prognosis in ACS patients with HbA1c < 7.0%. Second, we were unable to determine all clinical characteristics with an effect on 1,5-AG levels (e.g., renal glycosuria). Third, we did not perform the 75 g oral glucose tolerance test in patients who were not previously diagnosed with DM. This may have led to underestimation of the number of patients with DM. Finally, the levels of glycoalbumin were not measured in this study. However, a previous study reported that 1,5-AG was independently associated with de-novo coronary artery disease, whereas HbA1c and glycoalbumin were not independently associated with the disease [46]. In concordance with the previous study, multivariate Cox regression analysis showed that 1,5-AG but not HbA1c was significantly associated with long-term cardiac mortality in the present study. We believe that the 1,5-AG level, which is considered a clinical marker of postprandial hyperglycemia, may be a useful marker for risk stratification in patients with coronary artery disease. Further studies will be required to confirm these findings.

## Conclusion

Low 1,5-AG levels, which indicate postprandial hyperglycemia, predicted long-term cardiac mortality in ACS patients with HbA1c < 7.0%.

## Abbreviations

1,5-AG: 1,5-anhydroglucitol
ACS: acute coronary syndrome
CI: confidence interval
DM: diabetes mellitus
eGFR: estimated glomerular filtration rate
HbA1c: hemoglobin A1c
HR: hazard ratio
